# A new chromatographic approach for the simultaneous determination of tramadol, ibuprofen, and caffeine in a novel fixed-dose combination tablet: an integrated framework to analytical sustainability and multimodal analgesia

**DOI:** 10.1186/s13065-025-01688-8

**Published:** 2025-12-14

**Authors:** Israa A. Wahba, Said A. Hassan, Ahmed S. Fayed, Sally S. El-Mosallamy

**Affiliations:** 1 Postgraduate Program in Pharmaceutical Analytical Chemistry, Faculty of Pharmacy, Cairo University, Cairo, 11562 Egypt; 2 Pharmaceutical Analytical Chemistry Department, College of Pharmaceutical Sciences and Drug Manufacturing, Misr University for Science and Technology, 6th of October City, Giza, Egypt; 3 Pharmaceutical Analytical Chemistry Department, Faculty of Pharmacy, Cairo University, Kasr-El Aini Street, Cairo, 11562 Egypt

**Keywords:** Multimodal analgesia, Caffeine, HPLC, Ibuprofen, Rational polypharmacy, Sustainability, Tramadol

## Abstract

Multimodal analgesia and rational polypharmacy have emerged as modern pain management strategies, offering synergistic pain relief while mitigating the adverse effects of high-dose monotherapy. A novel antinociceptive fixed-dose combination tablet containing tramadol, ibuprofen, and caffeine exemplifies this approach by integrating a centrally acting weak opioid tramadol, a peripherally acting nonsteroidal anti-inflammatory drugs ibuprofen, and a central nervous system stimulant adjuvant caffeine into a single formulation. In the present work, the first HPLC method for the concurrent quantification of tramadol, ibuprofen and caffeine in bulk powder and tablet dosage forms was developed and validated to support the quality control of this innovative multimodal analgesic fixed-dose combination. The optimal chromatographic separation was achieved using a Zorbax SD-C8 column (150 × 4.6 mm, 5 µm) as the stationary phase. The mobile phase was composed of acetonitrile: 0.01 M phosphate buffer pH 5.0 (35.0: 65.0, v/v), delivered at a flow rate of 1.0 mL/min with UV detection at 220.0 nm. The separation was achieved within 8.0 min, with linearity ranges of 1.0–45.0 μg/mL for tramadol, 1.0–25.0 μg/mL for ibuprofen, and 1.0–30.0 µg/mL for caffeine. The suggested method was validated according to the ICH guidelines and successfully utilized for the quantitative determination of tramadol, ibuprofen and caffeine in bulk powder, laboratory-prepared mixtures and pharmaceutical formulation. The proposed method exhibited outstanding performance for determination of the three cited drugs, characterized by high accuracy (ranging from 100.42% to 100.82%) and excellent precision (< 2% RSD). The sustainability profile of the method was assessed using the Analytical eco-scale, Analytical GREEnness metric, Green analytical procedure index, Blue Applicability Grade Index, White analytical chemistry, and Violet Innovation Grade Index to evaluate the sustainability, applicability, and innovative potential of the proposed method. The method combines analytical rigor, operational simplicity, and sustainable design, setting a new benchmark for analytical support of rational polypharmacy products.

**Supplementary Information:**

The online version contains supplementary material available at 10.1186/s13065-025-01688-8.

## Introduction

Pain is a complex, multifactorial phenomenon and remains among the most frequent causes for seeking medical attention worldwide. The International Association for the Study of Pain (IASP) defines it as an “unpleasant sensory and emotional experience associated with, or resembling that associated with, actual or potential tissue damage” [1]. Pain may be classified as acute or chronic, somatic or visceral, and manifests in various anatomical regions. Acute pain typically arises suddenly in response to trauma or inflammation, and, if inadequately treated, may progress to chronic pain, causing functional impairment and reduced quality of life [2].

To address this widespread clinical concern, the World Health Organization (WHO) introduced the analgesic ladder, a stepwise approach beginning with non-opioid analgesics (paracetamol and nonsteroidal anti-inflammatory drugs (NSAIDs) for mild pain, progressing to weak opioids like tramadol for moderate pain, and finally to strong opioids for severe pain [3]. Although effective, this model has evolved toward multimodal strategies that integrate drugs with complementary mechanisms of action.

Multimodal analgesia, or rational polypharmacy, combines analgesics from different pharmacological classes to enhance efficacy and minimize adverse effects [4]. This strategy acknowledges the intricate pathophysiology of pain and the limitations of monotherapy. Numerous reports have demonstrated the advantages of opioid–NSAID combinations as a safer and more effective foundation for pain control [5], and have shown reduced tolerance and dependence compared with opioid monotherapy [6]. Against the backdrop of the ongoing opioid crisis, combining opioids with non-opioid agents such as NSAIDs and central nervous system (CNS) stimulants has emerged as a promising strategy to maintain efficacy and improve safety [6].

The concept of combining centrally acting agents with peripherally acting drugs—supplemented with adjuvants such as caffeine (CAF)—is based on the well-established pharmacological principle of “Multimodal Analgesia”. This strategy is particularly well-suited for bridging the therapeutic gap between moderate and severe pain, where monotherapy often proves insufficient [7]. Among the most promising combinations in this context is the triple regimen of an opioid, an NSAID, and CAF, exemplified by novel fixed-dose formulations containing tramadol (TRM), ibuprofen (IBU), and CAF. Each component contributes distinct pharmacological effects, and the combination allows lower individual doses to achieve effective pain relief while reducing dose-related side effects [8, 9].

**Fig. 1:**
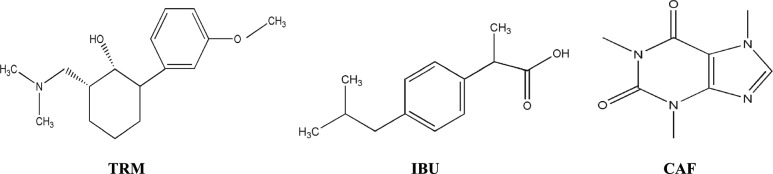
Chemical structure of the compounds under study

Tramadol HCl (Fig. 1), chemically designated as trans-(6)-2-[dimethylamino] methyl)-1-(3-methoxyphenyl) cylcohexanol hydrochloride [10], is a centrally acting analgesic with a weak µ-opioid receptor agonist effect [11]. Chronic pain of either malignant or nonmalignant origin and postoperative pain can be effectively managed with TRM. It represents a suitable therapeutic alternative for patients at risk of cardiopulmonary compromise or in situations where non-opioid analgesics are contraindicated [12]. TRM undergoes hepatic metabolism to its O-desmethyl metabolite (M1), which exhibits approximately 200-fold greater affinity for the opioid receptor than the parent drug [12]. Considering its abuse potential, the FDA has classified tramadol as a Schedule IV controlled substance [13]. Several techniques have been reported for the determination of TRM either alone or in the presence of other drugs, including spectroscopic [14], chromatographic [15, 16], and electrochemical methods [17].

Ibuprofen (Fig. 1), chemically known as 2-(4-Isobutylphenyl) propanoic acid [10], belongs to the NSAID class and exerts its analgesic, antipyretic, and anti-inflammatory effects by inhibiting both cyclooxygenase enzymes (COX-1 and COX-2). IBU is one of the most widely used over-the-counter analgesics for both adults and children prescribed to relieve several types of pain and to reduce inflammation and nociceptor sensitization [18]. Ibuprofen is indicated for the management of numerous conditions, including inflammatory and rheumatoid disorders, mild-to-moderate pain, fever, dysmenorrhea, and osteoarthritis. It is also regarded as a preferable alternative to aspirin and indomethacin because of its association with fewer side effects [18]. IBU has been determined in bulk powder or in combination with other drugs using spectroscopic [19, 20], chromatographic [21, 22], and electrochemical methods [23].

Caffeine (Fig. 1), chemically named 3,7-Dihydro-1,3,7-trimethyl-1H-purine-2,6-dione [10], is a well-known CNS stimulant that reduces fatigue and enhances alertness [24]. Although CAF has little to no analgesic effect when administered alone [25], it acts synergistically as an adjuvant when co-administered with NSAIDs, enhancing antinociceptive effects through mechanisms such as adenosine receptor antagonism and improved drug absorption [26, 27]. It functions effectively as an adjuvant in combination analgesic drugs due to its rapid absorption and distribution. Furthermore, a synergistic analgesic interaction has been reported between CAF and TRM [28]. CAF has been determined alone or in combination with other drugs using spectroscopic [29, 30], chromatographic [31, 32] and electrochemical methods [33].

This combination is particularly advantageous in acute and postoperative pain contexts, where rapid onset and multi-dimensional pain coverage are required. While TRM provides central analgesia, IBU targets peripheral inflammation, and CAF enhances analgesic efficacy while counteracting opioid-induced sedation [11]. The fixed-dose formulation contains 50 mg TRM, 400 mg IBU, and 32 mg CAF per tablet, typically administered as two tablets daily for effective multimodal pain control. Despite their potential, fixed-dose combinations (FDCs) of opioid, NSAID, and CAF remain scarce globally, with most marketed products confined to Latin America, the Middle East, and parts of Asia. Their limited presence in Western markets reflects a gap between pharmacological potential and regulatory adoption.

In this context, the analytical development and clinical evaluation of FDCs comprising opioids, NSAIDs, and CAF represents an important frontier in rational polypharmacy. These triple combinations align with WHO recommendations on multimodal analgesia, offering a safe, effective, and practical approach for managing diverse pain conditions. A variety of analytical techniques—chromatographic, spectroscopic, and electrochemical—have been employed for pharmaceutical analysis and quality control (QC), with chromatographic methods remaining the most reliable and widely adopted [34–38].

Despite being individually monographed in the BP [39], no official or literature-reported method currently exists for the simultaneous determination of TRM, IBU, and CAF in a triple formulation. However, various analytical techniques have been proposed for these drugs only in their binary combinations [40, 41].

Therefore, the aim of this work is to support the multimodal analgesia approach by developing and validating the first chromatographic method for the concurrent determination of TRM, IBU, and CAF in bulk powder and pharmaceutical formulations. This method aims to facilitate efficient QC and promote the broader adoption of this promising combination in the pharmaceutical market toward safe rational polypharmacy. Furthermore, the method's environmental sustainability was assessed using several greenness evaluation tools, including the Analytical Eco-Scale, Analytical GREEnness metric (AGREE), Green analytical procedure index (GAPI), Blue Applicability Grade Index (BAGI), White Analytical Chemistry (WAC), and Violet Innovation Grade Index (VIGI).

## Experimental

### Instruments

HPLC separation was carried out using an Agilent 1260 Infinity Series LC with a 1260 Quaternary Pump and a variable wavelength detector (controlled via Chemstation 32 software) with an autosampler injector (1260 ALS). The separation was performed at room temperature using a Zorbax SD-C8 column (5 µm, 150 × 4.6 mm).

### Materials and reagents

The three studied drugs were kindly provided by EVA Pharma (Egypt), and their purity was assessed according to BP assay methods [39], found to be 100.04% ± 0.94, 99.13% ± 1.12, and 99.57% ± 0.85 for TRM, IBU, and CAF, respectively. PAIN MED® tablets, labeled to contain 50.0 mg TRM, 400.0 mg IBU, and 32.0 mg CAF were manufactured by INTERMED company (India) and are typically administered at a dose of two tablets per day.

HPLC-grade methanol and acetonitrile were purchased from Merck (Germany). The analytical-grade chemicals included potassium dihydrogen orthophosphate (Oxford, India), orthophosphoric acid (Biotech, Egypt), ethylene diamine (Biotech, Egypt) and double-distilled deionized water (Otsuka Cairo, Egypt).

Phosphate buffer (pH 5.0; 0.01 M) was prepared by dissolving 1.36 g of potassium dihydrogen orthophosphate in a 1000-mL volumetric flask, then filling up the volume with double-distilled deionized water. The pH was subsequently adjusted using either ethylenediamine or orthophosphoric acid [39].

### Standard solutions

Stock standard solutions of TRM, IBU, and CAF (1.0 mg/mL) were prepared in methanol. Working solutions of the three cited components (100.0 µg/mL) were prepared by further dilutions with the mobile phase solution (acetonitrile: phosphate buffer, pH 5.0; 35.0: 65.0, v/v).

### Chromatographic conditions

Chromatographic separation was conducted on Zorbax SD-C8 (150 × 4.6 mm, 5 µm) column. The mobile phase, acetonitrile: phosphate buffer (35.0: 65.0, v/v), pH 5.0, was filtered through a 0.45 µm membrane filter and degassed ultrasonically for 15.0 min. The flow rate (1.0 mL/min) was maintained throughout the analysis, and the UV detector was set at 220.0 nm. The injection volume was 10.0 μL, and all measurements were performed at ambient temperature.

### Procedures

#### Construction of the calibration curves

To establish calibration curves for TRM, IBU, and CAF in the ranges of 1.0–45.0, 1.0–25.0 and 1.0–30.0 µg/mL, respectively, precise aliquots were transferred from the working solutions of TRM, IBU, and CAF into three separate sets of 10-mL volumetric flasks. The volumes were brought up to the mark with the mobile phase. An aliquot of 10.0 μL from each concentration was injected into the analytical column, and separation was conducted as mentioned under chromatographic conditions. Calibration curves were then constructed by plotting the peak area versus drug concentration, and regression equations were computed.

#### Assay of laboratory-prepared mixtures

Aliquots with different ratios of TRM, IBU, and CAF were transferred from their corresponding working solutions into a series of 10-mL volumetric flasks. The volume of each solution was completed to the mark with the mobile phase, and the procedure outlined above was followed. The concentrations of each component were calculated using the corresponding regression equations.

#### Method validation

The proposed method was validated for linearity, accuracy, precision, sensitivity (LOD and LOQ), robustness, and specificity in compliance with the ICH Q2(R1) guideline [42]. Linearity was assessed as described under *Construction of the calibration curves*. Accuracy was evaluated at three concentration levels in triplicate covering the entire calibration range: 5.0, 16.0, and 30.0 μg/mL for TRM; 7.0, 17.0, and 22.0 μg/mL for IBU; and 5.0, 14.0, and 25.0 μg/mL for CAF. Dilutions were prepared from the respective working standard solutions (100.0 µg/mL) into separate series of 10-mL volumetric flasks, and volumes were completed with the mobile phase. Precision was determined as repeatability (intra-day) and intermediate precision (inter-day) using the same concentration levels as accuracy, analyzed in triplicate on the same day and over three consecutive days, respectively. Sensitivity was calculated according to ICH guidelines using the standard deviation of response (σ) and calibration slope (S): LOD = 3.3σ/S and LOQ = 10σ/S. Robustness was examined by deliberate variations in chromatographic conditions, including acetonitrile content (± 2%), flow rate (± 0.1 mL/min), and phosphate buffer pH (± 0.1).

#### Application to pharmaceutical dosage form

Ten tablets were accurately weighed and finely powdered. A quantity of the powder equivalent to one tablet was transferred to a 100-mL volumetric flask containing 15.0 mL of methanol. Following 20.0 min of sonication, the volume was made up to the mark with methanol. Subsequently, filtration was carried out using 0.45 μm Whatman filter paper. Appropriate dilutions were performed using the mobile phase. The general procedure previously mentioned under chromatographic conditions was applied to ascertain the concentrations of the three investigated drugs in the obtained dosage form solutions.

## Results and discussion

The present work introduces the first green HPLC method for the accurate, efficient, and eco-friendly determination of TRM, IBU and CAF in a fixed-dose tablet formulation. This triple combination offers a novel multimodal analgesia approach, aiming to deliver enhanced pain relief while minimizing adverse effects often associated with high-dose monotherapies.

### Method development and optimization

The main goal of the proposed method development was to achieve high-resolution separation of the three active pharmaceutical ingredients (APIs) in their FDCs, while ensuring sharp, symmetric peaks, short analysis time, and compliance with green analytical chemistry (GAC) principles. Several critical chromatographic parameters were systematically optimized, including column type, mobile phase composition, pH, flow rate, and wavelength selection.

Different columns were initially tested to evaluate their influence on separation efficiency and analysis time. Columns such as Zorbax X SB-C18 and Kromasil Phenyl yielded suboptimal results, exhibiting poor resolution and prolonged retention times, likely due to their inability to effectively accommodate the diverse physicochemical characteristics of the analytes: TRM, a moderately polar amphiphilic compound with basic amine and hydroxyl moieties (log P = 3); IBU, a highly lipophilic weak acid with a bulky hydrophobic side chain (log P = 4); and CAF, a small, polar, planar purine derivative (log P = − 0.07) [10]. The C18 column, driven primarily by hydrophobic interactions, retained IBU excessively while providing insufficient separation for the more polar TRM and CAF, resulting in broad or overlapping peaks and long retention time. The phenyl column, relying on π-π interactions, showed insufficient discrimination among these molecules may be due to similar aromaticity in their structures. Consequently, neither of these columns could effectively balance the retention, selectivity, speed, and peak shape required for simultaneous separation of this chemically diverse mixture. In contrast, the Zorbax SD-C8 column offered superior selectivity and balanced hydrophobicity, enabling better resolution for the moderately retained TRM and CAF, while also preventing excessive retention of IBU. The shorter alkyl chain (C8) provides less hydrophobic retention than C18, which is advantageous for mixtures with varying polarities. This translated into sharper, more symmetrical peaks, reduced analysis time, and excellent system suitability parameters, including theoretical plate number, resolution, and tailing factor (as shown in Table 1). Therefore, Zorbax SD-C8 was the optimal choice for this chromatographic system.

**Table 1:** Parameters required for system suitability tests of the proposed HPLC method

Parameters	CAF	TRM	IBU	Reference value [68]
Retention time (t_R_) (min)	2.10	3.80	7.40	–
Resolution (Rs)^a^	–	6.51	8.55	> 2
Selectivity factor (α)^a^	–	1.87	1.93	> 1
Tailing factor (T)	1.00	1.15	1.05	0.8–1.2
Capacity factor (K)	2.31	3.45	5.73	0 < K < 10
Column efficiency (N)	3363	3744	4863	˃ 2000
Height equivalent to theoretical plate (mm)	0.045	0.040	0.031	The smaller the value, the higher the column efficiency

^a^ Chromatographic resolution and selectivity factor are determined between each peak and the one preceding it

Different mobile phases with variant compositions between aqueous (water and phosphate buffer) and organic (methanol, acetonitrile, and ethanol) phases were tested to provide quick separation and improved performance of the chromatographic system. The organic solvent played a decisive role in achieving adequate elution. Among the organic modifiers tested, methanol and ethanol resulted in broad, poorly resolved peaks with elongated run times. In contrast, acetonitrile, owing to its stronger elution strength and lower viscosity, yielded sharper peaks and significantly reduced retention times.

The pH of the mobile phase played a pivotal role in modulating the ionization states and retention behavior of the analytes, particularly TRM and IBU, both of which are ionizable under various pH ranges. IBU, a carboxylic acid with pKa ≈ 4.8–5.2 [10, 43–45], exists predominantly in its unionized (neutral) form at acidic pH, enhancing hydrophobic interactions with the stationary phase and thereby increasing retention. At alkaline pH, IBU becomes fully ionized (negatively charged), resulting in reduced retention due to decreased hydrophobic affinity. On the other hand, TRM, a weak base with pKa ≈ 9.4, is fully protonated at acidic pH and remains largely in its cationic form across most tested pH values. In strongly acidic conditions, its hydrophilic, charged state reduced retention and contributed to overlapping peaks with polar CAF. As the pH increased toward neutrality, partial deprotonation led to slightly improved retention, but alkaline conditions did not significantly enhance resolution and instead increased overall run time. CAF, which is a neutral molecule across the studied pH range (as a weak base with very low ionization), showed minimal pH sensitivity in terms of retention time. Therefore, a moderately acidic pH of 5.0 proved to be the optimal compromise, ensuring that IBU remains largely unionized and well-retained, TRM exhibits moderate retention with reduced peak overlap, and CAF maintains consistent elution. This condition facilitated baseline resolution, improved peak symmetry, and a balanced retention profile across all three analytes, supporting reliable quantification in a single, efficient chromatographic run. Consequently, the optimal mobile phase was identified as acetonitrile: 0.01 M phosphate buffer (pH 5.0) in a 35:65 v/v ratio.

Several flow rates (0.8–1.5 mL/min) were evaluated. A flow rate of 1.0 mL/min was optimal, offering a balance between peak resolution and short analysis duration. Several wavelengths (210.0, 220.0, and 230.0 nm) were examined, and 220.0 nm was found to be the optimal detection wavelength, providing sufficient sensitivity for all the cited drugs.

**Fig. 2:**
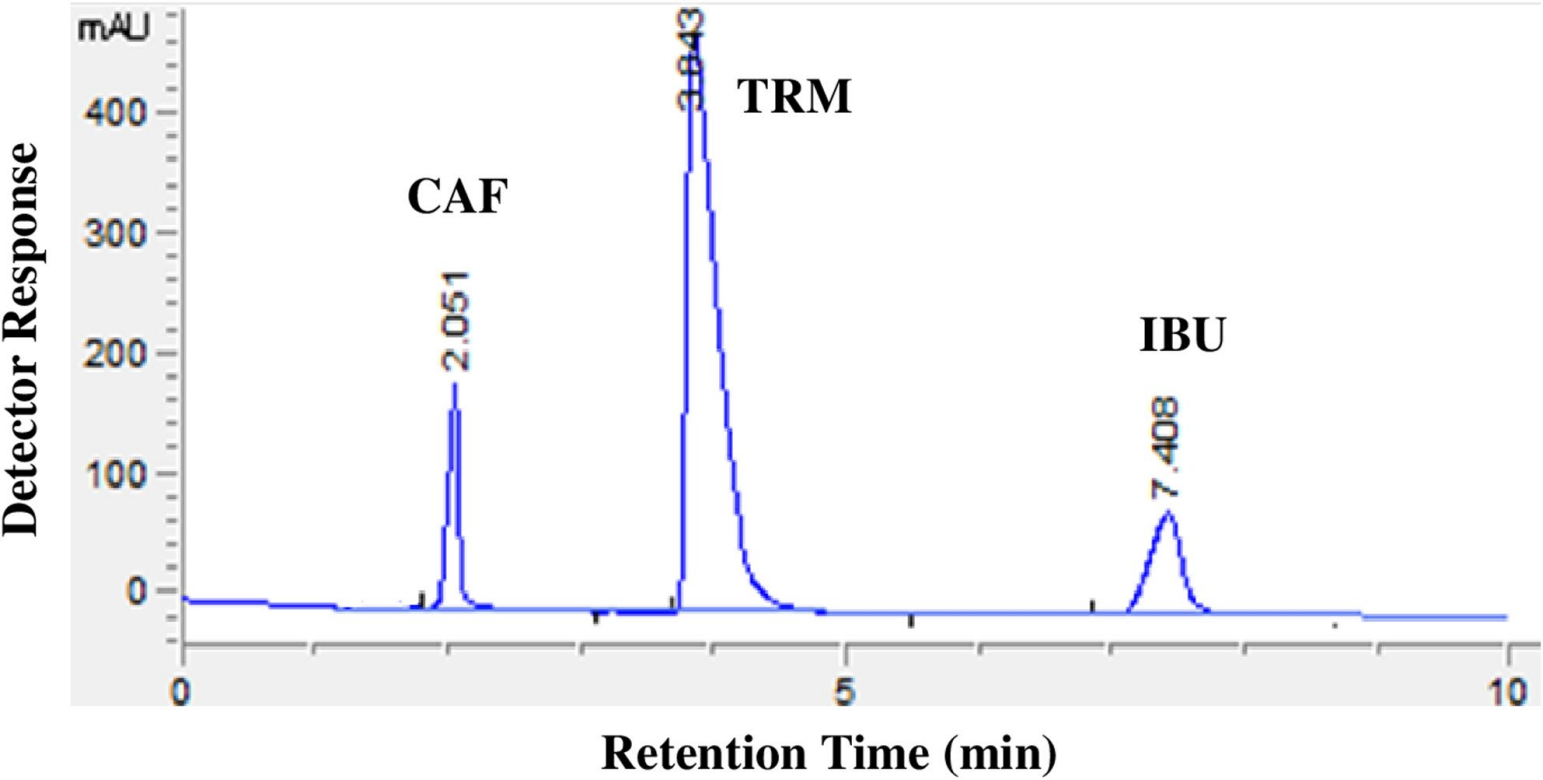
HPLC chromatogram of caffeine (10.0 μg/mL) (t_R_ = 2.1), tramadol (10.0 μg/mL) (t_R_ = 3.8) and ibuprofen (10.0 μg/mL) (t_R_ = 7.4), using a Zorbax SD-C8 (5 µm, 150 × 5.6 mm i.d.) column and isocratic elution of mobile phase composed acetonitrile: phosphate buffer pH = 5.0 at a ratio of 35.0: 65.0 V/V, at a flow rate of 1.0 mL/min, and detection at 220.0 nm

Under these optimized conditions, the chromatogram (Fig. 2) demonstrates excellent resolution of CAF (t_R_ = 2.1 min), TRM (t_R_ = 3.8 min), and IBU (t_R_ = 7.4 min) with clear baseline separation and short run time (< 8.0 min), which is ideal for routine pharmaceutical QC. System suitability parameters (Table 1) confirmed the reliability and efficiency of the developed method. Resolution (Rs) between successive peaks exceeded the USP criterion of Rs > 2, achieving 6.51 (TRM/CAF) and 8.55 (IBU/TRM), indicating excellent peak separation. Selectivity factors (α) of 1.87 and 1.93 support the method’s capability to distinguish analytes with similar retention behavior. Tailing factors for all drugs ranged between 1.0 and 1.15, confirming good peak symmetry. Capacity factors (K') fell within ideal limits, ensuring appropriate retention for all compounds. Column efficiency (N) was excellent exceeding 2000 for the three analytes with very small corresponding plate heights, affirming the high efficiency separation.

### Method validation

The proposed method was validated in compliance with the ICH Q2(R1) guideline [42], with systematic evaluation of key performance parameters as detailed in Table 2.

**Table 2:** Parameters and validation data for the determination of CAF, TRM, and IBU by the proposed HPLC method

Parameters	CAF	TRM	IBU
Range (μg/mL)	1.0 -30.0	1.0 -45.0	1.0- 25.0
Regression equation	y = 25.774*x* + 1.5404	y = 22.392*x* + 64.154	y = 28.523*x* + 12.345
SD of slope	0.15	0.17	0.22
RSD% of slope	0.57	0.77	0.78
Variance of slope	0.02	0.03	0.05
F statistic	30,597.47	16,847.29	16,542.91
Significance F	6.41 × 10^−9﻿^	2.11 × 10^−8^	2.19 × 10^−8^
SD of intercept	2.65	4.90	3.42
Correlation coefficient (r)	0.9999	0.9998	0.9998
LOD^a^ (μg/mL)	0.29	0.31	0.25
LOQ^a^ (μg/mL)	0.87	0.95	0.76
Accuracy (R% ± SD)^b^	100.80 ± 0.70	100.82 ± 1.08	100.42 ± 1.15
Repeatability (RSD%)^c^	1.24	1.21	1.38
Intermediate precision (RSD%) ^d^	1.55	1.67	1.79

^a^ LOD and LOQ were calculated using the following equations:$LOD=3.3\upsigma /\mathrm{S}$and$LOQ=10\upsigma /\mathrm{S}$(σ is the residual standard deviation, *S* is the slope)

^b^ Mean recovery percentage and standard deviation (*n* = 3) of three concentration levels

^c^ Intraday precision: three replicates at each of three concentrations within the same day

^d^ Interday precision: three replicates at each of three concentrations over three consecutive days

Good linearity was obtained for all three analytes within the concentration ranges of 1.0–45.0 μg/mL for TRM, 1.0–25.0 μg/mL for IBU, and 1.0–30.0 μg/mL for CAF. As summarized in Table 2, the calibration models exhibited excellent correlation coefficients (r ≥ 0.9998). The regression equations demonstrated low standard deviations and variances of the slopes (≤ 0.05), with RSD% of slope < 0.8%, ensuring stability of detector response. Similarly, the standard deviations of the intercepts were minimal, supporting model consistency. The high F-statistics (1.65–3.06 × 10^4^) and correspondingly low significance F values (10^−8^–10^−9^) indicate strong regression fits and statistically meaningful calibration lines. These statistical indicators collectively verify the model’s robustness and predictive reliability across all studied concentration levels. The inclusion of advanced linearity diagnostics was performed to ensure the analytical model fulfills both regulatory and statistical quality criteria [46].

Accuracy was in triplicate at three concentration levels for the three analytes. The method demonstrated excellent recovery, with mean values ranging from 99.86 to 100.88%, confirming the trueness of the method. Precision, evaluated as repeatability and intermediate precision at three concentration levels produced RSD% values well below 2.0%, confirming the method’s precision, as shown in Table 2 and Supplementary Table S1.

Sensitivity was demonstrated by low limits of detection (LOD) and limits of quantification (LOQ), calculated as per the ICH guidelines. The LOD values were 0.29 µg/mL for CAF, 0.31 µg/mL for TRM, and 0.25 µg/mL for IBU, indicating the method’s ability to detect trace amounts of the analytes with high confidence. The corresponding LOQ values were all close to 1.0 µg/mL, supporting precise quantification even at low concentrations.

Robustness was carefully checked via intentionally varying key chromatographic parameters: acetonitrile percentage (± 2%), flow rate (± 0.1 mL/min), and phosphate buffer pH (± 0.1). As detailed in Table 3, none of these variations caused significant deviations in system suitability parameters and recovery percentages (R%). The method retained its performance integrity under all tested conditions, with RSD% values for robustness tests consistently below 2%, affirming its resilience against small procedural fluctuations.

**Table 3:** Robustness assessment of the investigated method for determination of CAF, TRM, and IBU

Parameters		CAF	TRM	IBU
		RSD%^a^		
Acetonitrile (%) 35.0 ± 2%	Rs^b^	**–**	1.60	1.27
	tR	1.68	1.73	1.35
	K	1.30	1.62	1.18
	T	1.16	1.20	1.45
	R%	1.79	1.54	0.95
Phosphate buffer pH 5.0 ± 0.1	Rs^b^	–	1.41	1.79
	tR	1.24	1.18	1.53
	K	1.39	1.62	1.02
	T	1.53	1.67	1.90
	R%	1.41	1.62	1.15
Flow rate 1.0 ± 0.1 mL/min	Rs^b^	––-	1.63	1.83
	tR	1.43	1.65	1.89
	K	1.24	1.59	1.75
	T	1.51	1.46	1.41
	R%	1.19	1.75	1.74

^a^ RSD% for each system suitability parameter at the three specified conditions

^b^ Resolution is determined between each peak and the one preceding it

Specificity was confirmed by the method’s ability to selectively resolve and quantify the analytes in the presence of each other and in various ratios. Analysis of laboratory-prepared mixtures (Table 4) yielded satisfactory R% in the range 98.33–101.96% for the three analytes. These values confirm that the method is specific and accurate across a wide range of concentration ratios, reflective of real-world formulations. Importantly, resolution values greater than 2.0, as shown in Table 1, further underscore the method's capability to achieve complete baseline separation for the ternary mixture.

**Table 4:** Determination of CAF, TRM, and IBU in laboratory-prepared mixtures by the proposed HPLC method

Conc. (μg/mL)			Recovery %^a^		
CAF	TRM	IBU	CAF	TRM	IBU
1.76	2.76	22.00	101.96 ± 1.18	101.12 ± 1.30	101.73 ± 1.56
1.92	3.00	24.00	101.54 ± 1.38	98.70 ± 1.59	98.91 ± 1.57
10.00	10.00	12.00	99.27 ± 1.40	98.92 ± 1.37	100.29 ± 1.52
10.00	15.00	15.00	99.27 ± 1.23	101.43 ± 1.27	101.25 ± 1.33
7.00	20.00	22.00	98.33 ± 1.26	99.37 ± 1.25	98.74 ± 1.43
Mean ± SD			100.07 ± 1.58	99.91 ± 1.28	100.18 ± 1.34

^a^ Average of three determinations

### Assessment of method sustainability

The principles of Green Analytical Chemistry (GAC) advocate the replacement of conventional, resource-intensive analytical techniques with methods that minimize environmental burden, occupational hazards, and waste generation while maintaining analytical performance [47]. However, modern thinking in analytical science has evolved beyond “pure greenness,” recognizing that sustainability requires a broader, multidimensional view that integrates not just environmental considerations, but also practical applicability, analytical performance, and innovation potential [48]. The principles of GAC are increasingly being applied across various analytical techniques, including spectrophotometry [49–51], spectrofluorimetry [52, 53], electrochemistry [54], HPLC [55–58] and TLC [59–61].

Accordingly, the developed HPLC method for TRM, IBU, and CAF was evaluated across an ascending framework: beginning with greenness tools (Eco-Scale, AGREE, GAPI), moving to practical applicability (BAGI), expanding into composite sustainability (White Analytical Chemistry, WAC), and finally addressing forward-looking innovation (VIGI). This progression demonstrates how the method not only reduces its ecological footprint, but also aligns with real-world implementation, holistic sustainability, and emerging technological frontiers.

#### Analytical eco-scale

The Analytical Eco-Scale is a semi-quantitative tool widely used for assessing the environmental impact of analytical methods. It operates on a 100-point ideal green score, deducting “penalty points” for factors such as hazardous reagent use, energy consumption, occupational risk, and waste production [62]. As shown in Table S2 (Supplementary Materials), the suggested HPLC method achieved a penalty point score of 86, classifying it as an “excellent green analysis”. Only moderate deductions were made for the use of acetonitrile, as no halogenated solvents or excessive hazardous reagents were involved. It should be noted that orthophosphoric acid and ethylenediamine were used only as trace pH modifiers (< 0.1 mL per liter) for fine adjustment of the phosphate buffer. Their negligible volumes do not significantly impact the greenness profile and were therefore not considered in the formal assessment tools. This high score reflects the method’s minimal toxicological footprint, moderate solvent consumption, and operational safety.

#### Analytical GREEnness metric (AGREE)

While Eco-Scale provides a numeric snapshot, AGREE offers a more nuanced greenness evaluation, integrating the 12 principles of GAC into a single pictogram. Each of the twelve segments represents one principle (e.g., waste minimization, energy efficiency, safety), with colors ranging from deep red (non-compliant) to dark green (fully compliant) [63].

**Fig. 3:**
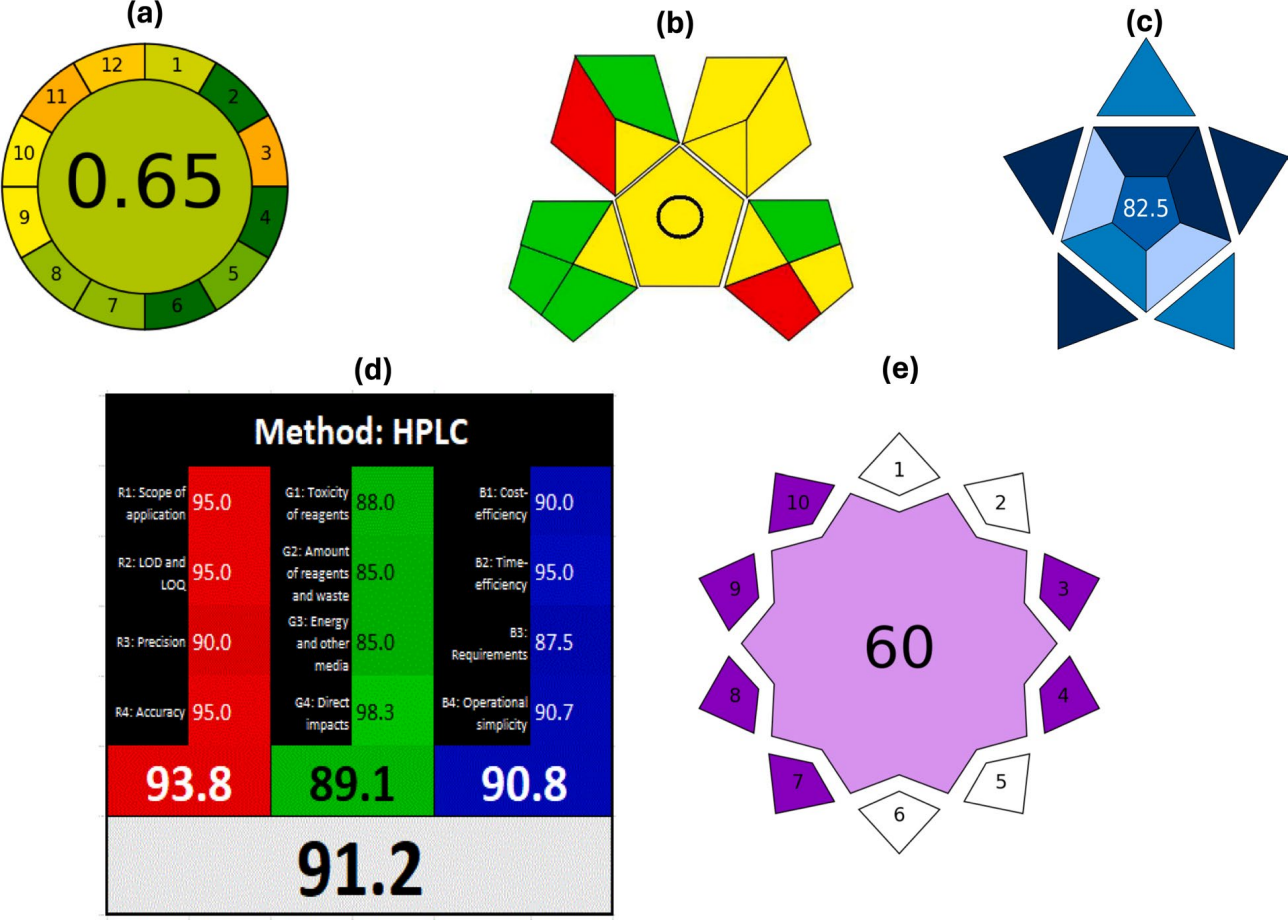
Sustainability assessment for the proposed method, **a** AGREE, **b** GAPI, **c** BAGI, **d** RGB12, and **e** VIGI

The method scored 0.65 (Fig. 3a), indicating strong overall ecological compatibility. The pictogram showed mostly green and yellow zones, with the orange sectors attributed to the at-line nature of HPLC and the effect of using non-green acetonitrile in the mobile phase. Despite this, the method demonstrates low environmental impact across key criteria.

#### Green analytical procedure index (GAPI)

Greenness profile assessment was comprehensively supplied by applying the GAPI tool. It is concerned with all aspects of analytical methodology, including sampling, sample preparation, chemicals used, instruments used, and the method of analysis overall [64]. The GAPI pictogram features a pentagram with 15 segments, color-coded green, yellow, or red [64]. Fewer red areas signal lower environmental burden. For the proposed HPLC method (Fig. 3b), green and yellow sectors dominated, with only minor red zones linked to solvent usage and at-line sample handling. This indicates that most procedural stages, including instrumentation, analysis, and waste management, are environmentally sustainable.

#### Blue applicability grade index (BAGI)

While greenness is important, an analytical method must also be practically applicable in real-world labs. A novel assessment tool, BAGI (Blue Applicability Grade Index), focuses mostly on evaluating the practicality and applicability of analytical methodologies [65]. BAGI offers a comprehensive assessment of an analytical method's perks and drawbacks in terms of ease of use, time efficiency, cost, and equipment requirements. The intensity of blue color in each section indicates how well the approach performs in that particular area. A deeper blue shade denotes greater efficiency and sustainability, whereas paler blue tones reflect weaker performance [65].

Within this investigation, the adopted HPLC approach yielded a remarkable BAGI score of 82.5 (Fig. 3c), well above the 60-point threshold considered acceptable for routine lab use. Our investigated method displays deeper blue hues in multiple sectors, including instrumentation, reagent consumption, and analysis time, demonstrating its minimal requirement for advanced instruments, fewer chemicals and superior efficiency. This score confirms that the method is not just green, but highly implementable and operator-friendly, a crucial consideration for QC labs.

#### White analytical chemistry (WAC)

White Analytical Chemistry (WAC) offers a framework for incorporating sustainability concepts into analytical techniques. Red, green, and blue light beams combine to produce the white color, which indicates the coherence and synergy of the analytical, ecological, and practical traits, according to an analysis of whiteness using the RGB12 model. The three parts of the RGB12 model combines analytical performance (Red), ecological impact (Green), and practical applicability (Blue) into a unified “whiteness” score [66].

Using the RGB12 model, each of the 12 sub-criteria (4 red, 4 green, 4 blue) is scored from 0 to 100. Figure 3d illustrates that the suggested method obtained a total whiteness score of 91.2. This balanced “white” profile confirms that the method is not only environmentally responsible but also analytically powerful and practically viable, bridging the gap between ideal green design and industrial needs.

#### Violet innovation grade index (VIGI)

Finally, beyond sustainability and applicability, innovation must be addressed. The Violet Innovation Grade Index (VIGI) tool was newly created in 2025 especially to measure and illustrate innovation in analytical techniques. It evaluates cutting-edge features like automation, interdisciplinarity, digital integration, and regulatory compliance among a total of ten distinct factors. Each parameter is assigned a score between 1 and 10, and the total of these individual outcomes is the ultimate VIGI assessment [67]. The method earned a VIGI score of 60 (Fig. 3e), indicative of considerable originality but with room for further innovation. The method scored well for methodology soundness, regulatory compliance, and potential integration into wider QC frameworks, but lower for manual sample preparation, data processing, innovative reagents, and miniaturization.

### Application to dosage form and statistical comparison

The proposed chromatographic method was successfully applied to the analysis of tablet formulation containing TRM, IBU, and CAF. The method demonstrated excellent selectivity and accuracy for the simultaneous determination of the three analytes within the tablet matrix (Table 5). Method accuracy and the absence of matrix interference were further confirmed by performing a standard addition recovery study, the results of which are summarized in Table 5. The obtained recoveries were satisfactory, confirming that common excipients did not interfere with the determination of any of the analytes.

**Table 5:** Determination of TRM, IBU, and CAF in tablet formulation using the proposed method and application of standard addition technique

	CAF			TRM			IBU		
Found %^a^ ± SD	99.41 ± 1.76			100.05 ± 1.65			99.70 ± 1.56		
Standard addition technique	Taken^b^ (μg/mL)	Added (μg/mL)	Recovery (%)	Taken^b^ (μg/mL)	Added (μg/mL)	Recovery (%)	Taken^b^ (μg/mL)	Added (μg/mL)	Recovery (%)
	9	4.5	101.94	10	5	101.62	8	4	99.28
		9	100.80		10	100.40		8	101.22
		18	99.61		20	98.97		16	98.07
Mean ± SD			100.78 ± 1.17			100.33 ± 1.33			99.53 ± 1.59

^a^ Average of five determinations of tablet dosage form

^b^ Different dilution for each drug to accommodate the added standard within the linearity range of the method

Furthermore, the results produced by the developed HPLC method were statistically compared with those obtained using the corresponding official pharmacopeial titrimetric assays [39]. As shown in Table 6, the calculated *t*- and *F*-values did not exceed the critical limits at the 95% confidence level, demonstrating that there was no significant difference between the proposed and official methods regarding accuracy, precision, or variance. These findings confirm that the developed method is reliable and suitable for routine quality control of this fixed-dose combination in bulk powder and tablet form.

**Table 6:** Statistical comparison for the results obtained by the proposed method and the official methods for the analysis of TRM, CAF and IBU

Parameters	CAF		TRM		IBU	
	HPLC	Official method ^a^	HPLC	Official method ^a^	HPLC	Official method ^a^
Mean R% (n = 3)	99.25	100.04	100.18	100.15	100.23	100.43
SD	0.977	1.321	1.142	1.316	1.069	1.266
Variance	0.954	0.954	1.305	1.733	1.144	1.603
n	5	5	5	5	5	5
F-test (6.388)	1.830	–	1.328	–	1.402	–
Student’s t-test (2.306)	1.068	–	0.040	–	0.269	–

^a^ Pharmacopeial titrimetric methods [39]

## Conclusion

The main goal of this work was to develop and validate the first chromatographic method for the simultaneous determination of tramadol, ibuprofen and caffeine in their novel fixed-dose combination tablet. The proposed method achieved fast, robust, and selective chromatographic separation using an environmentally considerate mobile phase, with analysis completed in under 8 min. Validation of the method was conducted in accordance with the ICH guidelines, confirming that the investigated method provides a reliable, sensitive, and accurate approach suitable for routine analysis of the studied drugs in pure bulk powder and finished pharmaceutical formulations. Beyond analytical performance, the method was rigorously assessed for sustainability, practicality, and innovation potential using a suite of modern evaluation tools, including the Analytical Eco-Scale, AGREE, GAPI, BAGI, WAC, and VIGI indices. High scores across these metrics confirm that the method is not only analytically sound, but also eco-friendly, operationally applicable, and forward-looking. Overall, this work sets a new benchmark for analytical support of multimodal analgesic fixed-dose combination, providing a validated, sustainable, and practical tool for quality control laboratories and paving the way for broader adoption of rational polypharmacy products in pain management.

## Supplementary Information

Supplementary Material 1.

## Data Availability

The authors declare that the data supporting the findings of this study are available within the paper and supporting materials.

## Abbreviations

AGREE: Analytical GREEnness metric
BAGI: Blue Applicability Grade Index
CAF: Caffeine
FDC: Fixed-dose combination
GAPI: Green analytical procedure index
HPLC: High-performance liquid chromatography
IBU: Ibuprofen
ICH: International council for harmonization
NSAIDs: Nonsteroidal anti-inflammatory drugs
TRM: Tramadol
VIGI: Violet Innovation Grade Index
WAC: White analytical chemistry
