# Supplementary material for: A new chromatographic approach for the simultaneous determination of tramadol, ibuprofen, and caffeine in a novel fixed-dose combination tablet: an integrated framework to analytical sustainability and multimodal analgesia
Source: BMC Chem. 2025 Dec 14;20(1):13. doi: 10.1186/s13065-025-01688-8 (PMC12821319; doi:10.1186/s13065-025-01688-8)
Supplement: Supplementary file 1 — Supplementary Material 1. [file 13065_2025_1688_MOESM1_ESM.pdf]

Supplementary Materials for
A New Chromatographic Approach for the Simultaneous Determination of Tramadol, Ibuprofen, and Caffeine in a Novel Fixed-Dose Combination Tablet: An Integrated Framework to Analytical Sustainability and Multimodal Analgesia

Israa A. Wahba [a,b]*, Said A. Hassan [c]*, Ahmed S. Fayed [c], Sally S. El-Mosallamy [c]

[a] Postgraduate program in Pharmaceutical Analytical Chemistry, Faculty of Pharmacy, Cairo University, Cairo 11562, Egypt.

[b] Pharmaceutical Analytical Chemistry Department, College of Pharmaceutical Sciences and Drug Manufacturing, Misr University for Science & Technology, 6th of October City, Giza, Egypt.

[c] Pharmaceutical Analytical Chemistry Department, Faculty of Pharmacy, Cairo University, Kasr-El Aini Street, Cairo 11562, Egypt.

*** Corresponding authors:**

Israa A. Wahba

E-mail: esraa.adel@must.edu.eg

Tel.: +201066885046

Said A. Hassan

E-mail: said.hassan@pharma.cu.edu.eg

Tel.: +201000994542

Table S1. Repeatability, intermediate precision, and accuracy of the proposed HPLC method for the determination of TRM, IBU, and CAF.

Drug	Concentration (µg/mL)	Precision		Accuracy (R% ± SD) [c]
		Repeatability (RSD%) [a]	Intermediate precision (RSD%) [b]	
CAF	5	1.78	1.23	100.65± 0.79
	14	1.03	1.71	101.69± 0.32
	25	0.90	1.69	100.05± 0.99
TRM	5	1.37	1.83	100.17±1.38
	16	0.88	1.98	101.58 ± 0.47
	30	1.37	1.18	100.71±1.39
IBU	7	1.31	1.79	101.08± 0.77
	17	1.53	1.83	100.26± 1.14
	22	1.30	1.74	99.93± 1.55

[a] Intraday (*n* = 3), three concentration levels for each compound repeated three times within the same day.

[b] Interday (n = 3), three concentration levels for each compound repeated three times in three different days.

[c] Mean (*n* = 3) of three concentration levels covering the specified range.

Table S2. Analytical eco-scale assessment for the proposed HPLC method

Hazard	Penalty points
	Proposed HPLC method
**Reagents**	
Acetonitrile	8
Phosphate buffer	0
Water	0
**Instrument**	
Energy (> 0.1kWh per sample)	1
Occupational hazard	0
Waste	5
**Total PPs**	Σ 14
**Analytical Eco-scale score**	86 excellent green analysis

Analytical eco-scale score = 100 (the ideal score of green analytical method).
Analytical eco-scale score >75 (an excellent green analysis).
Analytical eco-scale score 50–75 (the green analysis is acceptable).
Analytical eco-scale score <50 (the green analysis is inadequate).
